# Comparative genomic analysis of regulation of anaerobic respiration in ten genomes from three families of gamma-proteobacteria (Enterobacteriaceae, Pasteurellaceae, Vibrionaceae)

**DOI:** 10.1186/1471-2164-8-54

**Published:** 2007-02-21

**Authors:** Dmitry A Ravcheev, Anna V Gerasimova, Andrey A Mironov, Mikhail S Gelfand

**Affiliations:** 1 Lomonosov Moscow State University, Department of Bioengineering and Bioinformatics, Moscow, 119992, Russia; 2 Institute for Information Transmission Problems, Moscow, 127994, Russia; 3 State Scientific Center GosNIIGenetika, Moscow, 113545, Russia

## Abstract

**Background:**

Gamma-proteobacteria, such as *Escherichia coli*, can use a variety of respiratory substrates employing numerous aerobic and anaerobic respiratory systems controlled by multiple transcription regulators. Thus, in *E. coli*, global control of respiration is mediated by four transcription factors, Fnr, ArcA, NarL and NarP. However, in other Gamma-proteobacteria the composition of global respiration regulators may be different.

**Results:**

In this study we applied a comparative genomic approach to the analysis of three global regulatory systems, Fnr, ArcA and NarP. These systems were studied in available genomes containing these three regulators, but lacking NarL. So, we considered several representatives of Pasteurellaceae, Vibrionaceae and *Yersinia *spp. As a result, we identified new regulon members, functioning in respiration, central metabolism (glycolysis, gluconeogenesis, pentose phosphate pathway, citrate cicle, metabolism of pyruvate and lactate), metabolism of carbohydrates and fatty acids, transcriptional regulation and transport, in particular: the ATP synthase operon *atpIBEFHAGCD*, Na^+^-exporting NADH dehydrogenase operon *nqrABCDEF*, the D-amino acids dehydrogenase operon *dadAX*. Using an extension of the comparative technique, we demonstrated taxon-specific changes in regulatory interactions and predicted taxon-specific regulatory cascades.

**Conclusion:**

A comparative genomic technique was applied to the analysis of global regulation of respiration in ten gamma-proteobacterial genomes. Three structurally different but functionally related regulatory systems were described. A correlation between the regulon size and the position of a transcription factor in regulatory cascades was observed: regulators with larger regulons tend to occupy top positions in the cascades. On the other hand, there is no obvious link to differences in the species' lifestyles and metabolic capabilities.

## Background

*Escherichia coli*, the best-studied representative of gamma-proteobacteria, can adapt to a wide variety of environmental conditions. One source of this capability is the presence of numerous aerobic and anaerobic respiratory systems. To adapt to different growth conditions, this bacterium alter the composition of their respiratory systems by changing the repertoire of substrate-specific dehydrogenases and terminal oxidoreductases. The concentration of each component is strictly regulated in order to optimize the respiratory chains according to the available substrates and the physiological needs of the cell.

In facultative anaerobe *E. coli*, regulation of respiration depends on the availability of electron acceptors, which are used in a specific order. Thus, molecular oxygen represses all other types of respiration and fermentation. Under anaerobiosis, nitrate, the most favorable electron acceptor in such conditions, represses other types of anaerobic respiration and fermentation. This control is effected by a variety of regulatory systems [1,2].

The first level of regulation is implemented via the Fnr protein. Fnr is an oxygen-sensitive transcription factor. Oxygen is sensed by iron-sulfur clusters formed by the N-terminal domain. Under anaerobic conditions, Fnr forms dimers that are capable of binding DNA, whereas in the presence of oxygen these clusters are reversibly destroyed and the dimers dissociate [3,4]. Thus, Fnr is active only under anaerobic conditions, when it activates genes necessary for the anaerobic metabolism and represses genes for the aerobic respiration [2,5]. In *E. coli*, Fnr is the top regulator of respiration, as it controls the expression of genes for other transcriptional factors [6,7].

Another transcription factor regulating respiration is the ArcA protein. ArcA is a part of the ArcA-ArcB two-component system, where ArcB is an inner membrane sensor protein. ArcB is belived to sense the redox state of ubiquinones: *in vitro*, the activity of this protein depends on the redox status of a ubiquionone soluble analog [8,9]. In the absence of corresponding electron acceptors, ArcB phosphorylates ArcA, enabling it to bind DNA [10,11]. Thus, under the anaerobic conditions, ArcA regulates expression of genes for respiration and central metabolism, and probably controls the switch between the respiration and fermentation metabolisms [5,12-14].

The next level of regulation is provided by two homologous transcription factors, NarL and NarP. These proteins are activated in the presence of nitrate and nitrite by two homologous sensor kinases NarX and NarQ. The duplicated two-component system allows for the fine tuning of the nitrate and nitrite respiration system to a dynamic ratio of two alternative substrates. NarX and NarQ respond differentially to nitrate and nitrite. Thus, in this subsystem three levels of the response specificity may be distinguished: (i) interaction between sensor proteins and respiratory substrates, (ii) interaction between sensor proteins and transcription factors, and (iii) interaction between factors and their binding sites in DNA [15-18]. Upon activation, NarL and NarP activate genes for the nitrate and nitrate respiration and repress genes for other, less effective, pathways of anaerobic respiration [19-22].

Nevertheless, in some gamma-proteobacteria, only the single NarQ-NarP system was found [23], accompanied by reduction of the nitrate and nitrite respiratory system, and the presence of only the periplasmic respiratory system. So, in most gamma-proteobacteria the fine tuning of the system is not required and a single NarQ-NarP system is sufficient for the control of the nitrate and nitrite respiration [24].

Although the physiology and regulation of respiration in *E. coli *has been studied in considerable depth, the regulation in other organisms is poorly understood. Moreover, in other representatives of gamma-proteobacteria, for example, in Haemophilus influenzae, regulation of respiration looks to be quite different from *E. coli *[25,26].

Previously, comparative genomic analyses were performed separately for Fnr [27], ArcA [28], and NarP [24] in a small number of microorganisms. Here we report the results of a comparative genomic analysis of the respiration regulation in multiple genomes belonging to several representatives of the three families of gamma-proteobacteria. Although the NarL-DNA interactions have been studied in experiment [29-32], the existing methods for prediction of transcription factor binding sites do not allow for the reliable identification of candidate NarL sites. Because of that, we analyzed the organisms containing only the NarP regulator. In such organisms, we studied three structurally different, but functionally related Fnr, ArcA and NarP systems.

## Results

### Structure of the Fnr, ArcA and NarP regulogs

The procedure described in "Methods" was applied to the analysis of regulation by Fnr, ArcA and NarP. All genes predicted to form regulogs (orthologous regulons) were classified according to their functions.

In comparison with previous studies, where single regulatory systems had been studied in several gamma-protoeobacterial genomes [24,27,33], here we predicted additional regulatory interactions. These predictions were made possible by the use of total pairwise comparison and detailed simultaneous analysis of multiple regulatory systems. Here we consider in detail only the relevant predictions. A summary of these predictions is given in Table 1, whereas more detailed data about site prediction, including information about operon structure changes, are shown in additional files 1 and 2.

#### Respiration

One of the most significant results was the identification of conserved candidate sites upstream of the *atpIBEFHAGCD *operon. This operon encodes all subunits of the ATP synthetase complex, the key component of the oxidative phosphorylation [34]. Experimental data about regulation of the *atp *operon in *E. coli *are conflicting. In particular, expression of the *atp *operon was claimed to be independent of Fnr or ArcA [35]. On the other hand, 1.5- to 8-fold increase of the expression level of various *atp *genes in the *fnr *mutant *E. coli*, as compared to the wild-type strain, was shown using a microarray assay [36]. Similarly, 1.9- to 14-fold increase of the expression level of various *atp *genes was shown in the *arcA *mutant strain [37]. Recently, candidate Fnr and ArcA sites were observed upstream of the *atp *operon in *E. coli *K-12 genome; these sites were conserved in many Enterobacteriaceae genomes [Tsiganova and Ravcheev, unpublished observation]. In the present study, candidate ArcA sites were observed upstream of the *atp *operon in both *Yersinia *spp., and Fnr sites were detected in all Vibrionaceae. Thus, in at least some gamma-proteobacteria, expression of the ATP synthetase genes is controlled by the global regulators of respiration.

Candidate regulatory sites were found upstream of the *nqrABCDEF *operon encoding Na^+^-exporting NADH dehydrogenase [38]. Candidate Fnr-binding sites are conserved in all studied genomes, whereas ArcA sites have been found in all Vibrionaceae and Pasteurellaceae, but not in the *Yersinia *spp. The regulation by NarP seems to be specific for the Pasteurellaceae. Orthologs for the *nqr *genes were not found in *E. coli*, but such orthologs were detected in *Klebsiella pneumoniae *and various Vibrionaceae and Pasteurellaceae [38-43]. On the other hand, *E. coli *has an H^+^-exporting NADH dehydrogenase enzyme, encoded by the *nuo *operon, whose expression is regulated by the NarL and Fnr transcription factors [44,45]. No genes of the *nuo *operon demonstrate homology with the *nqr *genes. Orthologs of the *nuo *operon were found in the *Yersinia *genomes, but they were not preceded by candidate Fnr, ArcA or NarP binding sites. Thus, in the studied genomes, non-homologous displacement with a partial change of function leads to the sodium-dependent energetics used in conjunction with the proton-dependent one.

One more new regulog member is the *dadAX *operon. The first gene of this operon encodes the small subunit of respiratory D-amino acids dehydrogenase [46]. Candidate ArcA sites were detected in the *Yersinia *and Vibrionaceae genomes upstream of the *dadAX *operon. Thus, the use of D-amino acids as electron donors in some genomes may be controlled by ArcA.

An interesting example of taxon-specific regulation was observed for duplicated operons for trimethylamine N-oxide (TMAO) reductases and formate dehydrogenases. Two operons for TMAO reductases, *torCAD *and *torYZ*, were found in *E. coli*. [47,48]. The *torCAD *expression is known to be repressed by the NarL protein and activated by the transcriptional factor TorR [49,50], whereas *torYZ *is transcribed at a constant low level [47]. In the three Pasteurellaceae genomes, where the *torCAD *genes are absent, candidate sites for all three studied regulators were found upstream of the *torYZ *operon. In the Vibrionaceae genomes, the *torCAD *transcription is likely controlled by Fnr, whereas the *torYZ *expression seems to be regulated by NarP (see Table 1).

A similar situation was observed in *E. coli *for two formate dehydrogenase operons: the *fdn *operon is known to be regulated by the Fnr, NarL, and NarP proteins [51,52], whereas the expression of the *fdo *operon is constant [53]. Previously Fnr binding site was predicted upstream of the formate reductase operon in *Y. pestis *[27]. Careful analysis of the phylogenetical trees for the Fdn and Fdo proteins (additional file 3) revealed that both *Yersinia *spp. contain *fdo *but lack *fdn *genes. In both *Yersinia *spp. expression of the *fdo *operon is regulated by all three studied regulators. Further, regulation was predicted for the *fdhD *gene whose product is essential for the formation of the Fdn and Fdo protein complexes [54]. Accordingly, candidate regulatory sites upatream of *fdhD *were found only in genomes containing the *fdo *or *fdn *operon.

#### Central metabolism and fermentation

Previously, respiration-dependent regulation of some genes involved in the central metabolism was studied in experiment [5,55,56] and computationally [27,28]. Here, candidate regulatory sites were observed upstream of some more genes involved in the glycolysis, the gluconeogenesis, the pentose phosphate pathway, and the pyruvate and lactate metabolism: *sfcA*, *ppsA*, *pckA*, *eno*, *pgk*, *talB *and *aldB*. Unexpectedly, in the Pasteurellaceae, candidate NarP binding sites were found upstream of operons for glycolysis/gluconeogenesis and citrate cycle enzymes, such as *eno*, *pgk*, *mdh *and *sucABCD *(see Table 1).

#### Metabolism of carbohydrates

Regulation of sugar metabolism genes by the three considered regulators has not been shown experimentally. Here, conserved candidate sites for Fnr, ArcA or NarP were found upstream of some operons involved in the metabolism of various sugars. This is not surprising: indeed, the sugar metabolism is closely related to the central metabolism and sugars provide substrates for the energy production [57]. In particular, candidate sites were observed upstream of the operons *ptsHI-crr *and *mtlADR *(glucose- and mannitol-specific phosphotransferase systems, respectively [58]), *deoCABD *(degradation of pyrimidine deoxynucleosydes via deoxyribose-phosphates to acetaldehyde and glycerol-3-phosphate [59]), *nagB *(glucosamine-6-phosphate deaminase [60]), *gntXY *(gluconate transport system [61]), *malQ *(amylomaltase [62]), and *glgBXCAP *(glycogen biosynthesis and catabolism [63]).

An unusual situation was observed for the *glg *genes. According to criteria described in "Methods", these genes were assigned to a single operon in the *Yersinia *spp. In the Pasteurellaceae, the *glg *genes are preceded by the *malQ *gene that belongs to the candidate *malQ-glgBXCAP *operon. In both *Yersinia *spp., candidate Fnr sites were found upstream of the *glgB *gene, whereas in the Pasteurellaceae, Fnr and NarP sites were found upstream of the *malQ *gene. Site conservation despite operon reorganization shows that the regulation of the *glg *genes by respiratory factors is important for the bacterial cell.

However, the degree of conservation of candidate sites for the carbohydrate metabolism genes is relatively low compared to genes from other groups, the observed sites are often taxon-specific, and there are almost no cases of double or triple combinations. This also is not surprising, since regulation of carbohydrate transport and catabolism is extremely flexible and fast-evolving (O.Laikova, personal communication). On the other hand, each individual predictions should be considered as preliminary.

#### Fatty acids metabolism

The regulation of operons involved in the fatty acids metabolism was not described previously, although there were some indirect indications to the existence of such regulation [27,64]. Some of these operons have conserved binding sites and thus are likely to be regulated.

The homologous *fabBA *and *fadIJ *operons encode protein complexes for beta-oxidation of fatty acids. The FadBA protein complex is used in the fatty acids degradation in both aerobic and anaerobic conditions, whereas FadIJ is used predominantly during anaerobiosis [64,65]. Previously an effect of the *arcA *mutation on the *fadBA *operon expression was observed, although direct regulation was not shown [13]. Here, conserved candidate sites for Fnr and/or ArcA were found upstream of both these operons (Table 1).

The regulation was also predicted for the *fadD*, *acpP-fabF*, and *fadL *operons involved in the fatty acids metabolism and transport.

There are two possible explanations for the regulation of the fatty acids metabolism by the respiratory regulators. First, the fatty acids metabolism is closely related to the central metabolism, for example, through acetyl-CoA [66,67]. Second, different enzymes for the fatty acids oxydation are preferred under aerobic and anaerobic conditions [64].

#### Nucleotide reductases

Fnr is known to activate the *nrdDG *operon in *E. coli *under anaerobiosis, whereas expression of one more operon encoding nucleotide reductase, *nrdAB*, is uneffected by the Fnr protein in *E. coli *[68]. This situation is conserved in the *Yersinia *spp. and the Vibrionaceae. In contrast, in the Pasteurellaceae, candidate Fnr sites were found upstream of the *nrdAB *operon. However, in *H. ducreyi*, where the *nrdDG *operon is absent, no candidate sites were found upstream of *nrdAB*. So, the overall picture seems to be that at least one nucleotide reductase operon must be transcribed constantly.

#### Transport

Previously, regulation by at least one of the studied regulators was shown experimentally for a number of *E. coli *transporter operons, see references in Table 1. Here, we identified additional transporters in the studied regulogs and demonstrated changes in the regulatory interactions.

Previously regulation of dicarboxylate transporters genes *dcuA*, *dcuB *and *dcuC *was shown for *E.coli *(see references in Table 1). However, in other gamma-proteobacteria the regulation of these genes may be different. Thus, the regulation of the *dcuA *and *dcuB *genes is typical for the *Yersinia *spp. and Pasteurellaceae, whereas in Vibrionaceae the regulation was observed for *dcuC*.

#### Regulators of transcription

Prediction of regulation of genes encoding different transcription factors seems to be one of the most interesting results of this study. Previously, the *fnr*, *arcA*, *narP*, and *narQ *genes were shown to be regulated by respiratory regulators in *E. coli *(see references in Table 1).

Fis is one of the most abundant DNA architectural proteins. It regulates supercoiling of bacterial DNA [69]. Experimentally it was shown that, in *E. coli*, Fis together with Fnr and/or ArcA regulates expression of the *nrf *[70], *nir *[71], *ndh *[72], *adhE *[73], *yfiD *[74], *sdhCDAB-sucABCD *[75], *acnB *[76], and *narK *[77] operons. In *E. coli*, the *fis *gene is cotranscribed with *dusB *[78] and the structure of the *dusB-fis *locus is conserved in all studied genomes. In the Pasteurellaceae, candidate Fnr and ArcA sites were found upstream of the *dusB *gene. Thus, in these genomes *fis *transcription is likely to be controlled by the respiratory regulators. Since Fis participates in the control of expression of respiration genes, it is possible that Fnr and ArcA regulate expression of the *fis *gene to optimize the transcription control of these genes.

The *cpxRA *operon encodes a two-component regulatory system involved in the environmental stress response [79]. Some data point to cross-interactions between the CpxR-CpxA and ArcA-ArcB systems in *E. coli *[80]. In the Pasteurellaceae, conserved Fnr and ArcA sites were detected upstream of the *cpx *operon.

OxyR is the transcriptional factor for the oxidative stress response [81]. In all Pasteurellaceae, candidate ArcA sites were observed upstream of *oxyR *gene. Since ArcA is active as a transcription factor only in the absence of the oxygen, it may be possible that in these conditions it represses the transcription of the unneeded oxygen sensor gene *oxyR*. However, the regulatory logic in this case is not absolutely clear.

In the Pasteurellaceae, conserved Fnr sites were found upstrean of the *fur *gene, whereas in the Vibrionaceae, the *fur *gene has candidate sites for ArcA. The observation that the Fur protein, ferric uptake regulator [82], is controlled by the respiratory regulators is not unexpected, because iron is an obligatory component of most respiratory complexes [2] and the transcriptional factor Fnr itself [83].

### Other members of regulogs

In addition to the regulogs members described above, some genes were assigned to regulogs based on the formal criterion (see "Methods"), although their relevance to the respiration and central metabolism is not obvious. These genes and the candidate Fnr, ArcA, and NarP sites are shown in additional files 4 and 5. Although in the absence of a functional link, these predictions are somewhat weaker then the ones in the previous sections, we belive that they still warrant experimental verification.

### Divergons and false-positive predictions

In some cases we found sites in intergenic regions between two genes forming divergons. In such cases it is not immediately clear, which of the two divergently transcribed operons is regulated. These divergent operon pairs are *fadR/nhaB-dsbB *in most studied genomes, *yfiC/brnQ, torD/torR*, and *yhdWXYZ/VV12702 *in the Vibrionaceae, and *fkpA/slyX *in the *Yersinia *spp. and the Pasteurellaceae. The data about candidate sites upstream of divergent genes are present in additional file 5.

In other cases candidate regulog members in divergons were dismissed, as functional analysis made it clear which of two divergent operons was regulated. This category includes the *argR*, *ung*, *nfo *and *potABCD *operons (additional file 6).

## Discussion

### Composition of regulogs in different families

The above analysis shows that regulation often differs between bacterial families. To assess the relative role of each studied regulator in different families, we calculated the number of genes and operons belonging to each regulog in each studied family. As expected, the composition of regulogs turned out to be quite diverse.

**Figure 1:**
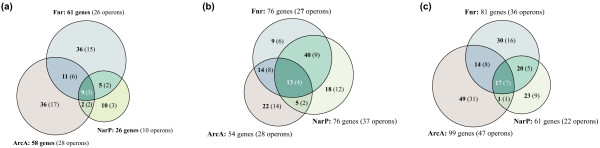
Regulogs composition in three families. **(a) **Enterobacteriaceae (*Yersinia *spp.); **(b) **Pasteurellaceae; **(c) **Vibrionaceae. The numbers of genes and operons in parentheses predicted to belong to the regulogs are shown.

For example, in the *Yersinia *spp., the NarP regulog is significantly smaller than in other studied families (Figure 1a). It is not surprising, given that in these bacteria the nitrate- and nitrite-respiration system is strongly reduced, and only nitrate, but not nitrite, is used as an electron acceptor [24]. Accordingly, in these organisms, the nitrate respiration plays a less prominent role, and the nitrate-sensing system also is less important as compared to other studied genomes.

In contrast, in Pasteurellaceae, the NarP regulog is somewhat enlarged in comparison with the other two taxons. One more feature of this group is the increased overlap between regulogs. For example, conserved sites for both Fnr and NarP regulators were found upstream of 13 operons containing 53 genes (Figure 1b).

In the Vibrionaceae, the ArcA regulog is extended as compared to the other groups. In this family, conserved ArcA sites were found upstream of 47 operons containing 99 genes (Figure 1c).

### Regulatory cascades

Group-specific regulatory cascades were predicted based on candidate sites upstream of the *fnr, arcA*, and *narP *genes.

**Figure 2:**
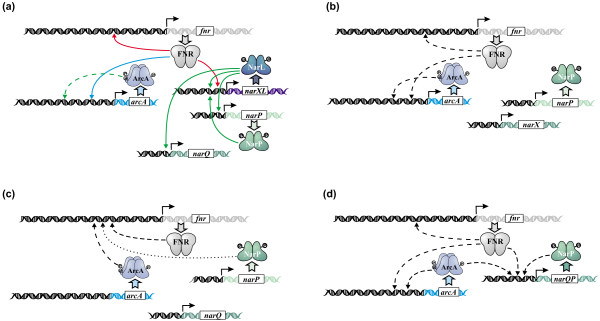
Respiration regulatory cascades in gamma-proteobacteria. **(a) ***E. coli*; **(b) ***Yersinia *spp.; **(c) **Pasteurellaceae; **(d) **Vibrionaceae. Experimentally determined regulation is shown by continuous arrows: green, activation; red, repression; blue, ambivalent regulation. Predicted regulation is shown by broken arrows: green, activation; black, type of regulation is not determined. Dotted arrows in **(c)**: sites were not found in *H. ducreyi*.

In *E. coli*, such cascades were analyzed experimentally (Figure 2a) [6,7,84]. There, the Fnr protein is the main regulator of the respiration that controls expression of genes for other regulators. Fnr represses transcription of the *narXL *operon [6] and regulates expression of the *arcA *gene [7]. Moreover, the *fnr *gene is autoregulated [6]. The *arcA *gene was predicted to be positively autoregulated [28]. The regulators of the nitrate and nitrate respiration, in turn, form a complicated regulatory network, where NarL activates expression of the *narXL, narP *and *narQ *operons, and NarP, in turn, activates the *narXL *transcription [84].

However, this situation is not conserved in the genomes of other gamma-proteobacteria. In the *Yersinia *spp., Fnr apparently regulates the *arcA *expression and both *fnr *and *arcA *genes are autoregulated (Figure 2b). No regulatory sites were found upstream of the *narP *and *narX *genes. Thus, in the *Yersinia *spp., Fnr is the main regulator of respiration, similarly to *E. coli*. However, unlike the situation in the latter, in the *Yersinia *spp., the nitrate- and nitrite-responsive regulatory system does not participate in the regulatory cascades.

Even more remarkable changes in the regulatory cascades were observed in the Pasteurellaceae. In most of these genomes, candidate sites for Fnr, ArcA and NarP were found upstream of the *fnr *gene (Figure 2c), whereas no conserved regulatory sites were observed for the *arcA *and *narP *genes. This suggests that in this family the role of Fnr in the regulatory cascades decreases in comparison with *E. coli*. Minor differences were seen in *H. ducreyi*, where no NarP sites were found upstream of the *fnr *gene (Figure 2c).

In the Vibrionaceae, autoregulation for all regulator genes, *fnr, arcA *and *narP*, was observed. The ArcA protein also seems to regulate the *narP *gene, whereas Fnr controls the expression of the *arcA *and *narP *genes (Figure 2d). Thus, in these organisms, Fnr again is the main respiration regulator, but the role of ArcA in the regulatory cascades is increased as compared to *E. coli*.

Thus, the *E.coli *pattern of regulatory cascades is not conserved in other gamma-proteobacteria, and the relative role of each regulator in the regulatory cascades differs between families. When more genomes become available, it might be possible to describe the evolution of this regulatory system in more detail.

### Family-specific regulatory interactions

**Figure 3:**
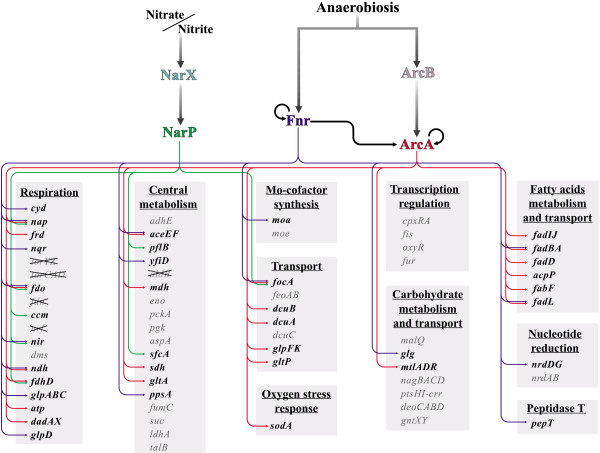
Regulatory interactions in *Yersinia *spp. Regulatory cascades are shown by thick black arrows. Predicted transcriptional regulation is shown by thin arrows, blue for Fnr, red for ArcA, and green for NarP.

**Figure 4:**
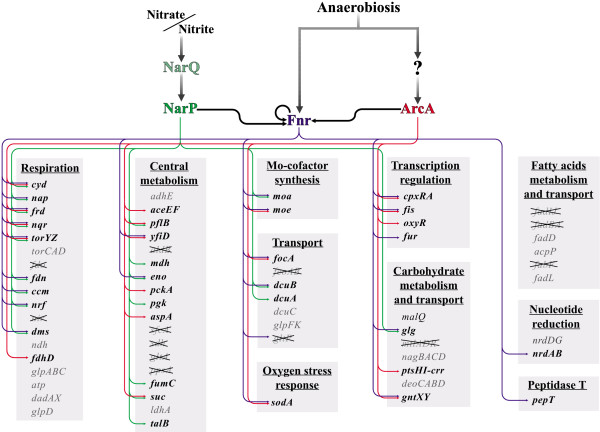
Regulatory interactions in Pasteurellaceae. Notation as in Figure 3.

**Figure 5:**
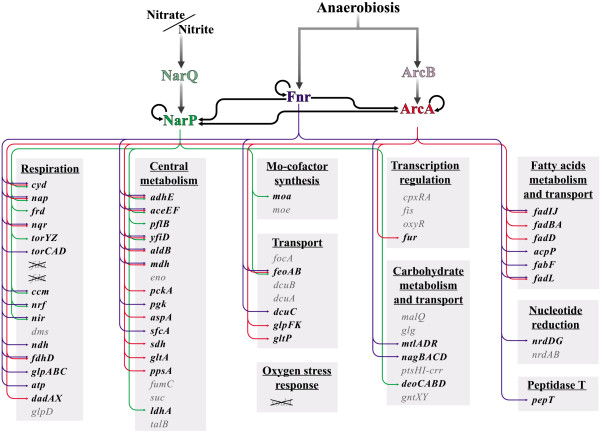
Regulatory interactions in Vibrionaceae. Notation as in Figure 3.

In addition to differences in the regulatory cacades, there are major differences in the functional content of the respiration regulogs. These data are summarized in Figures 3, 4, 5.

#### Enterobacteriaceae (*Yersinia spp*.)

Two genomes of the *Yersinia *spp. were selected for this study because of the presence of a single regulator of the nitrate and nitrite respiration regulator NarP. These bacteria demonstrate a number of features that distinguish them from both *E. coli *and the two other studied families (Figure 3).

Most of these features relate to the nitrate and nitrite respiration. First of all, regulation is mediated by the unusual pair NarX-NarP, in contrast to other bacteria that have the pair NarQ-NarP (for details see below).

Another feature of the *Yersinia *spp. regulation is simplification of the regulatory cascades (see above). In comparison with *E. coli*, only the Fnr-ArcA cascade is conserved, whereas nitrate and nitrite responsive proteins seem to be excluded from cascades. This has an effect on the NarP regulog: in the respiration subsystem, candidate sites were found only upstream of genes essential for the nitrate respiration using formate as an electron donor (*nap, ccm, fdo, fdhD*), and upstream of the *nir *operon, whose products protect the cell from the toxic nitrite.

On the contrary, the Fnr and ArcA regulogs seemed to be quite similar to the corresponding regulogs in other studied genomes.

#### Pasteurellaceae

These bacteria demonstrate considerable changes in the regulatory cascade structure (see above). In the organisms from this group, Fnr loses the role of the main regulator.

A conspicious feature is the absence of orthologs for the ArcA-activating sensor ArcB (data not shown). Such situation was observed in another gamma-proteobacterium, *Shewanella oneidensis *MR-1 [85]. It is possible that the role of the ArcA-activating protein is played by CpxA that is homologous to ArcB. Regulation of the *cpxRA *operon by Fnr and ArcA also points to this possibility.

A specific feature of the Pasteurellaceae is also the existence of downstream cascades, as several transcription factor-encoding genes, *cpxRA, fis, oxyR *and *fur*, are preceded by conserved candidate sites. All of these operons, except *fur*, have no candidate sites in other studied genomes. One possible explanation might be that drastic re-structuring of the regulatory cascades during evolution of the Pasteurellaceae required additional fine tuning of the respiratory metabolism.

The increased role of the NarP transcriptional factor in the regulatory cascades is consistent with the expansion of the NarP regulog (Figure 4). This expansion is realized in different ways. For example, upstream of the *cyd *and *nqr *operons, NarP sites emerged in addition to the existing Fnr and ArcA ones. In the case of the *mdh, eno *and *suc *operons, we observe a change in the regulatory interactions, where NarP candidate sites appear instead of Fnr of ArcA sites seen in other families. Finally, NarP sites upstream of the *eno *and *talB *genes appear in the Pasteurellaceae, while there are no sites for the studied factors in other genomes.

#### Vibrionaceae

In these genomes, the Fnr role as the main regulator is conserved, but the role of the ArcA in the regulatory cascades slightly increases.

Again, this agrees with the expansion of the ArcA regulog, as there appear ArcA sites upstream of genes of central metabolism (Figure 5). Conserved candidate ArcA binding sites were also found upstream of other genes (additional file 4).

Ten analyzed representatives of gamma-proteobacteria occupy different ecological niches: *H. ducreyi *is an obligate pathogen [86], *Y. pestis*, *Y. enterocolitica*, *P. multocida*, *A. actinomycetemcomitans*, H. influenzae, *V. vulnificus*, *V. parahaemolyticus *and *V. cholerae *are facultative pathogens [87-94], and *V. fischeri *is a squid symbiont [95]. However, there are no obvious links between the bacterial lifestyle and specific regulatory interactions or regulog composition.

### Variability of the nitrate and nitrite responsive regulatory system

Comparison of three global transcription regulators, Fnr, ArcA and NarP, demonstrates that the NarP-dependent regulation is the most flexible.

As we could not construct a NarL-site recognition rule, we considered only genomes containing NarP but not NarL. In all these genomes, the cytoplasmic nitrate respiratory system is absent and all reactions for the reduction of nitrate through nitrite to ammonium must occur in the periplasm. For this purpose, the proteins encoded by *nap, ccm *and *nrf *are sufficient. On the other hand, the genes for the cytoplasmic nitrate reduction, *narGHJI *and *narK*, are absent. In some studied genomes, the *Yersinia *spp. and most of the Vibrionaceae, the *nir *operon also is conserved [24]. This operon is used for the cytoplasmic nitrite reduction to ammonium, neutralizing toxic nitrite and providing ammonium as a substrate for the nitrogen assimilation [2]. Without the use of the cytoplasmic nitrite reduction, there is no need to select between the more effective, but also more dangerous, nitrate reduction in the cytoplasm, and the less effective but safer periplasmic reduction. Thus, all studied organisms do not require duplicated two-component regulatory systems and use the single pair NarP-NarQ.

In the *Yersinia *spp., an unusual sensor-regulator pair, NarX-NarP, was observed (additional file 7) [24]. However, in these genomes the sensor protein lacks the cysteine cluster typical for NarX from other species [23,24]. Thus, we can suppose that NarX from *Yersinia *spp. acts as NarQ.

Another interesting result was the observation of conserved candidate NarP sites upstream of genes for aerobic respiration, glycolysis/gluconeogenesis and citrate cycle, *mdh, eno, pgk, sucABCD *and *nqr *in Pasteurellaceae, and *cydAB *in the Pasteurellaceae and the Vibrionaceae. Regulation of genes for the aerobic metabolism by NarQ-NarP system may be explained by the fact that the NarQ sensor protein is sensitive to aeration [96].

## Conclusion

Previously the regulation of respiration in the gamma-proteobacteria was well studied only in *Escherichia coli *and two experimental approaches prevailed. The first one was studying the regulation of a particular operon by multiple factors. The second approach was the analysis of multiple genes controlled by a specific regulatory system in one genome. With sequencing of complete genomes and development of comparative genomic methods, extensive analysis of a complex regulatory system in multiple genomes has become possible.

Here we applied a comparative genomic technique to the analysis of global regulation of respiration in numerous gamma-proteobacterial genomes. We analyzed three structurally different but functionally related regulatory systems.

One of the advantages of the applied technique is the possibility to study genomes for which no experimental data are available. It also allows one to find new members of regulons and to detect taxon-specific features of regulation. Thus, the saturation of the taxonomic space by complete genome sequences increases the opportunities for the application of comparative genomics.

In this article and supplementary materials we present the data about candidate sites, both conserved and non-conserved, in multiple genomes. These data are interesting from the point of view of evolution and also may be of use for experimental biology. At that, prediction of the specific sites upstream of particular operons facilitates the work of experimentalist in finding new members of regulons.

In the particular case of the respiration regulation, we were able to predict not only taxon-specific members of regulons, but also taxon-specific changes in regulatory cascades. We demonstrated considerable changes of regulatory interactions in different families and relative stability of the metabolic and regulatory systems taken as a whole. Similar observations were made in studies of dissimilatory metabolism of nitrogen oxides [97], iron homeostasis [98] and nitrogen fixation (work in progress). Overall, existence of a stable system core, re-wiring of regulatory cascades accompanied by shuffling of the regulators, and existence of taxon-specific periphery seem to be common features of all complex regulatory systems. At present, it is not clear what drives these changes. We have observed a correlation between the regulon size and the position of a regulator in regulatory cascades: regulators with larger regulons tend to occupy top positions in cascaeds, and thus re-wiring of cascades is accompanied with extension of regulons. On the other hand, there is no obvious link to differences in the species' lifestyles, metabolic capabilities, etc.

## Methods

### Genomes

Respiration was studied in ten genomes of organisms from three bacterial families:

Enterobacteriaceae (*Yersinia pestis *KIM, YP; *Yersinia enterocolitica *type 0:8, YE), Pasteurellaceae (*Pasteurella multocida *Pm70, PM; *Actinobacillus actinomycetemcomitans *HK1651, AA; *Haemophilus influenzae *Rd, HI; *Haemophilus ducreyi *35000H, HD), and Vibrionaceae (*Vibrio vulnificus *CMCP6, VV; *Vibrio parahaemolyticus *RIMD 2210633, VP; *Vibrio cholerae *O1, VC; *Vibrio fischeri *ES114, VF). All studied organisms contain FNR, ArcA and NarP, but lack NarL.

The complete genome sequences of *Y. pestis *[99], *P. multocida *[100], *H. influenzae *[101], *H. ducreyi *[GenBank:AE017143], *V. vulnificus *[102], *V. parahaemolyticus *[103], *V. cholerae *[104], and *V. fischeri *[105] were downloaded from the GenBank database [106]. The complete sequence of *Y. enterocolitica *was taken from the Sanger Institute web site [107]. The complete sequence of *A. actinomycetemcomitans *was taken from the University of Oklahoma's Advanced Center for Genome Technology web site [108].

### Comparative genomics of family-specific regulation

To describe three interacting regulatory systems, comparative genomic approaches were used [109]. Candidate sites were identified in upstream regions of annotated genes, including hypothetical ones. A gene was considered a putative member of a regulog (a group of regulated orthologs) [110], if it had an upstream candidate site in several genomes. Site search was performed in gene upstream regions, -500 to +100 nucleotides relative to the gene start, excluding coding regions of upstream genes. The recognition profiles (nucleotide weight matrices) [109] for Fnr [27], ArcA [28] and NarP [24] were described previously. The recognition profiles and sequence logos are shown in additional files 8 and 9, respectively.

The cutoff profile scores for ArcA and FNR sites were set using known *E. coli *sites from the dpinteract database [111]. All *E. coli *FNR-binding sites passed the threshold 3.75, and all *E. coli *ArcA binding sites, but one outlier (upstream of the gene *pflA*), scored above the used threshold 4.00. With these parameters about 600 genes per genome had candidate sites in upstream regions. The cutoff for candidate NarP binding sites (3.50) was defined as the minimum score of candidate NarP sites upstream of the nitrate and nitrite respiration genes (*nap, nrf and ccm*) in all studied genomes. This cutoff produced about 300 genes per genome.

Clearly, most candidate sites at this stage were false positives, but the threshold could not be made stricter without loss of true sites. Anyhow, after the filtration procedure described below the number of candidate regulog members significantly decreased. Only few genes with no obvious functional link to the studied systems remained, and in most cases this was caused by sites occurring upstream of divergently transcribed genes. These observations demonstrate that the filtration procedure seems to be sufficiently effective to remove most false positives. On the other hand, each individual prediction should be considered as preliminary. Another caveat is that "orphan" sites (species-specific sites upstream of genes not regulated by other considered factors) cannot be identified by the applied procedure.

Possible operon structure was taken into account as follows. Genes were assumed to belong to one operon if (i) they were transcribed in the same direction, (ii) the intergenic distances did not exceed 200 nucleotides, and (iii) two previous conditions were met in genomes from different families. These conditions are known to perform well in operon prediction [111].

As the regulation of respiration had not been experimentally analyzed in any of the studied organisms, we developed a technique based on exhaustive pairwise comparison of genomes within taxonomic groups. For the Pasteurellaceae and the Vibrionaceae, a gene was assigned to a regulog if candidate regulatory sites were found upstream of an operon containing this gene in at least three genomes of organisms from the same family. For the *Yersinia *spp., a gene was considered to be a member of a regulog if candidate sites were found in both *Y. pestis *and *Y. enterocolitica *genomes. Such sites were called "conserved sites". Sites that did not satisfy these requirements were called "nonconserved". Further, if a site for one regulator was found upstream of a particular gene in one taxon, we searched for sites for all three regulators upstream of this gene and its orthologs in all studied genomes.

This approach allowed us to find new members of regulogs and to describe taxon-specific features of regulation. Its limitation is that it allows one to detect only the fact of regulation but, due to the absence of mapped promoters in the studied genomes, does not predict the type of regulation, such as repression or activation.

### Programs

The search for candidate sites was done using Genome Explorer [112]. Orthologs were determined as pairs of best bi-directional hits [113] identified by Genome Explorer [112]. Multiple sequence alignments and phylogenetic trees were done using ClustalX [114]. Search for homologs of studied genes in sequence databases was done using the BLAST program [115] with E-value cutoff e^-20^.

## Authors' contributions

AAM and MSG designed and coordinated the project. DAR and AVG carried out the genome analysis. DAR and MSG drafted the manuscript. All authors have read and approved the final manuscript.

## Supplementary Material

Additional File 1 Predicted regulatory interactions. For genome abbreviations see "Methods". Candidate sites are shown by letters: F – Fnr sites; A – ArcA sites, N – NarP sites; conserved sites are shown by capital letters, non-conserved ones by lower-case letters (for details see "Methods"). Absence of sites is shown by dashes. Absent genes are shown by zeros. Cases when the operon structure is not conserved are denoted by superscripts, and the structures of the corresponding operons are described in detail in additional file 2 using the matching superscripts. Click here for file

Additional File 2 Observed changes in the operon structures. For genome abbreviations see "Methods". The superscripts correspond to the superscripts in additional file 1. Click here for file

Additional File 3 Phylogenetic trees for FdnG/FdoG (a), FdnH/FdoH (b) and FdnI/FdoI. The trees were constructed by the neighbour-joining method. The expected fraction of amino acid substitutions is indicated for branches longer than 0.05. Genome abbreviations: EC – *Escherichia coli*, ST – *Salmonella typhi*, EO – *Erwinia carotovora*, YP – *Yersinia pestis*, YE – *Y. enterocolitica*, PM – *Pasteurella multocida*, AA – *Actinobacillus actinomycetemcomitans*, HI – *Haemophilus influenzae*. The analysis of the trees indicates that the most parsimonious evolutionary scenario is the duplication of the fumarate dehydrogenase operon in the ancestral Enterobacteria and further species-specific loss of the Fdn copy in the *Yersinia *lineage. In any case, it is clear that the fumarate dehydrogenase of the *Yersinia *spp. is Fdo. Click here for file

Additional File 4 Additional predicted regulatory interactions. "For genome abbreviations see "Methods". Candidate sites are shown by letters: F – Fnr sites, A – ArcA sites, N – NarP sites; conserved sites are shown by capital letters, non-conserved ones, by lower-case letters (for details see "Methods"). Absence of the corresponding sites is shown by dashes. Absent genes are shown by zeros. Superscripts point to changes in operon structures described in detail in additional file 5. ^#^: operon forms a divergon with an another one." Click here for file

Additional File 5 Additional observed changes in the operon structures. For genome abbreviations see "Methods". Superscripts coincide with the corresponding superscripts in additional file 4. Click here for file

Additional File 6 False-positive predictions. The last column lists the reasons to exclude the operons. For genome abbreviations see "Methods". Click here for file

Additional File 7 Phylogenetic trees for NarL/NarP (a) and NarX/NarQ (b). The trees were constructed by the neighbour-joining method. The expected fraction of amino acid substitutions is indicated for brances. The branches corresponding to *Yersinia *spp. proteins are shown with broken lines. Genome abbreviations: EC – *Escherichia coli*, ST – *Salmonella typhi*, EO – *Erwinia carotovora*, YP – *Yersinia pestis*, YE – *Y. enterocolitica*, PM – *Pasteurella multocida*, AA – *Actinobacillus actinomycetemcomitans*, HI – *Haemophilus influenzae*, HD – *Haemophilus ducreyi*, VV – *Vibrio vulnificus*, VP – *V. parahaemolyticus*, VC – *V. cholerae*, VF – *Vibrio fischeri*. Click here for file

Additional File 8 Position weight matrices (profiles) for Fnr (a), ArcA (b) and NarP (c) binding sites. Rows: nucleotides. Columns: Positions. Cells: positional nucleotide weights. Click here for file

Additional File 9 Sequence logos for the Fnr (a), ArcA (b) and NarP (c) binding sites. Horizontal axis, position in the binding site; vertical axis, information content in bits. The height of each column is proportional to the positional information content in the given position; the height of each individual symbol reflects its prevalence in the given position. Click here for file

## Figures and Tables

**Table 1:** Predicted regulatory interactions. For genome abbreviations see "Methods".

**Operon**	**Genome**									
	
	**YP**	**YE**	**PM**	**AA**	**HI**	**HD**	**VV**	**VP**	**VC**	**VF**
Respiratory enzymes										

atpIBEFHAGDC*	-A-	fAn	fan	-an	---	f--	F--	Fa-	F-n	F--
*cydAB*	Fan	Fa-	FA-	F-N	FAN	FAN	FAN	FAN	FAN	FAN
napFDAGHBC*	FAN	FAN	F-N	F-N	FaN	FaN	FAN	FAN	FAN	FAN
*ccmABCDEFGH*	f-N	--N	F-N	FaN	F-N	FaN	FaN	FaN	--N	F-N
nrfABCDEXFG*	**0**	**0**	F-N	F-N	FaN	FaN	FaN	F-N	**0**	FaN
*nirBDC-cysG*	F-N	F-N	**0**	**0**	**0**	**0**	F-N	F-N	**0**	F-N
*dmsABC*	f-n	**0**	F-N	F-N	F-N	**0**	**0**	fa-	**0**	fan
*torYZ*	**0**	--n	**0**	FAN	FAN	FAN	**0**	--N	--N	--N
torCAD*	**0**	**0**	fan	**0**	**0**	**0**	Fa-	F-n	Fa-	F-n
*frdABCD*	-A-	fA-	FAN	FAN	FAN	FAN	faN	--N	--N	faN
*fdoGHI*	FAN	FAN	FAn	FAn	-A-	**0**	**0**	**0**	**0**	**0**
*fdnGHI*	**0**	**0**				**0**	**0**	**0**	**0**	**0**
*fdhD*	-AN	-AN	fAn	fAn	-A-	-A-	**0**	---	**0**	**0**
*nqrABCDEF*	Fa-	F--	FAN	FAN	FA-	FAN	FA-	FA-	FA-	FAn
*ndh*	FA-	FAn	**0**	fA-	-An	fAn	F--	F--	F--	F--
*glpABC*	F--	Fa-	F--	---	F--	Fa-	Fa-	**0**	F--	F-n
*glpD*	F--	F--	**0**	f--	**0**	**0**	---	---	f--	fa-
*dadAX*	-A-	-A-	**0**	**0**	**0**	**0**	fA-	-An	-An	**0**

Molibdenum cofactor synthesis										

moaABCDE*	Fa-	F--	F--	F-N	F-N	FaN	--N	--N	--N	---
*moeAB*	---	f--	F--	FA-	FA-	FA-	---	---	---	-a-

Central metabolism and fermentation										

*pgk*	---	---	--N	---	faN	-aN	F--	F--	F--	F--
*eno*	---	-a-	F-N	FaN	F-N	F-	---	--n	---	---
*aldB*	**0**	**0**	**0**	**0**	**0**	**0**	FA-	FA-	FA-	FA-
*adhE*	fa-	---	f-n	f-n	**0**	---	FAN	FAN	FAN	FAN
pdhR-aceEF-lpdA*	FA-	FA-	-A-	-An	-A-	-An	FA-	F-N	FAN	FAN
*pflB*	--N	--N	-AN	-AN	-A-	f-N	-AN	--N	fA-	-AN
*yfiD*	Fa-	F--	FA-	FA-	FA-	-An	F-N	FAN	FAN	FAN
*mdh*	-A-	fA-	f-N	-aN	f-N	-aN	FA-	FA-	FA-	---
*pckA*	---	---	--n	-A-	fAn	fA-	-A-	-An	-A-	fAn
*ppsA*	F--	F--	**0**	**0**	**0**	**0**	-A-	fA-	---	-A-
*aspA*	f--	-a-	-A-	f--	-A-	-An	--N	--N	**0**	faN
*sfcA*	--N	faN	**0**	---	**0**	**0**	F--	F--	F--	F-n
*sdhCDAB*	-A-	-A-	**0**	**0**	**0**	**0**	-A-	fA-	fA-	-A-
*gltA*	-A-	-A-	-an	**0**	**0**	**0**	-A-	fA-	fA-	-A-
*fumC*	f--	---	-aN	--N	--N	f--	**0**	-a-	fa-	**0**
SucABCD*	---	---	-AN	-A-	fAN	fAN	---	---	---	-a-
*ldhA*	---	f--	**0**	---	-a-	**0**	-a-	--N	f-N	-aN
*talB*	f--	---	-aN	**0**	f-N	f-N	-a-	f--	---	---

Metabolism of carbohydrates										

*mtlADR*	fA-	-A-	---	**0**	**0**	**0**	F--	Fa-	F--	**0**
nagBACD*	---	---	fa-	---	---	--n	F--	F--	F--	F-n
*ptsHI-crr*	--n	---	-A-	-An	-A-	fA-	---	--n	-a-	--n
deoCABD*	f--	-a-	---	---	f--	f--	--N	--N	---	--N
malQ-glgBXCAP*	Fa-	F--	F-N	F-N	F-N	**0**	-an	f--	fa-	fan

Fatty acids metabolism										

*fadIJ*	-A-	-A-	**0**	**0**	**0**	**0**	FA-	FA-	F--	FA-
*fadBA*	FAn	FA-	**0**	**0**	**0**	**0**	-A-	fA-	-A-	fA-
*fadD*	fA-	-An	---	-a-	-an	f--	-A-	-A-	-A-	-A-
acpP-fabF*	-A-	-A-	fan	---	---	---	F--	F--	F--	---

Oxygen stress response										

*sodA*	-A-	-A-	FA-	FA-	FAn	FA-	**0**	**0**	**0**	**0**

Nucleotide reductases										

*nrdDG*	F--	F--	f--	-an	f--	**0**	F--	Fa-	F--	F--
*nrdAB*	--n	---	F-n	F--	Fa-	---	---	---	f--	f--

Transport										

*focA*	FAN	FAN	FA-	FA-	FA-	FA-	---	f--	---	fa-
*dcuB*	-A-	fA-	F-N	F-N	**0**	F-N	-a-	---	f--	fan
*dcuA*	-A-	fA-	--N	f-N	f-N	-a-	---	--n	---	--n
*dcuC*	**0**	f--	-a-	-a-	**0**	**0**	Fa-	F--	F--	Fa-
*gltP*	-An	fA-	**0**	**0**	**0**	**0**	fAn	-A-	-A-	fAn
*glpFK*	-An	-A-	f--	**0**	-an	fa-	-A-	-An	---	-An
*gntXY*	---	---	FA-	F--	FA-	FA-	---	---	---	--n
*fadL*	FA-	FA-	---	-a-	f--	-a-	FA-	-A-	FA-	FA-
*feoAB*	---	f--	**0**	**0**	**0**	**0**	FAN	FAN	-AN	FA-

Peptidase T										

*pepT*	F--	F--	F--	F--	F--	**0**	F--	Fa-	F--	Fan

Transcription regulators										

*fnr*	F--	F--	FAN	FAN	FAN	FA-	Fa-	Fa-	F--	F--
*arcA*	FA-	FA-	f--	---	f-n	-a-	FA-	FA-	FA-	FA-
*narQP*	-a-	--n	f--	--n	fan	-a-	FAN	FAN	FAN	FAN
*dusB-fis*	-a-	---	FA-	-A-	F--	FA-	---	---	---	f--
*cpxRA*	---	---	FA-	FA-	FA-	FAn	---	---	---	-a-
*oxyR*	---	---	-A-	-A-	-A-	fA-	---	f--	---	-a-
*fur*	---	---	F--	Fa-	F--	F--	-A-	-A-	-A-	-An

Candidate sites are shown by letters: F – Fnr sites; A – ArcA sites, N – NarP sites; conserved sites are shown by capital letters, non-conserved ones by lower-case letters (for details see "Methods"). Absence of sites is shown by dashes. Absent genes are shown by zeros. Cases when the operon structure is not conserved are denoted by asterics.
