# Supplementary material for: Comparative genomic analysis of regulation of anaerobic respiration in ten genomes from three families of gamma-proteobacteria (Enterobacteriaceae, Pasteurellaceae, Vibrionaceae)
Source: BMC Genomics. 2007 Feb 21;8:54. doi: 10.1186/1471-2164-8-54 (PMC1805755; doi:10.1186/1471-2164-8-54)
Supplement: Additional File 1 — Predicted regulatory interactions. For genome abbreviations see "Methods". Candidate sites are shown by letters: F – Fnr sites; A – ArcA sites, N – NarP sites; conserved sites are shown by capital letters, non-conserved ones by lower-case letters (for details see "Methods"). Absence of sites is shown by dashes. Absent genes are shown by zeros. Cases when the operon structure is not conserved are denoted by superscripts, and the structures of the corresponding operons are described in detail in additional file 2 using the matching superscripts. [file 1471-2164-8-54-S1.pdf]

Operon	Genome									
	YP	YE	PM	AA	HI	HD	VV	VP	VC	VF
Respiratory enzymes										
atpIBEFHAGDC	-A-[1a]	fAn[1a]	fan[1b]	-an[1b]	---[1b]	f--[1b]	F--[1a]	Fa-[1a]	F-n[1a]	F--[1a]
cydAB	Fan	Fa-	FA-	F-N	FAN	FAN	FAN	FAN	FAN	FAN
napFDAGHBC	FAN[2a]	FAN[2a]	F-N[2b]	F-N[2b]	FaN[2b]	FaN[2b]	FAN[2a]<br>f-n[2c]	FAN[2a]<br>f-n[2c]	FAN[2a]	FAN[2a]
ccmABCDEFGH	f-N	--N	F-N	FaN	F-N	FaN	FaN	FaN	--N	F-N
nrfABCDEXFG	**0**	**0**	F-N[3a]	F-N[3a]	FaN[3b]<br>-a-[3c]	FaN[3b]<br>F--[3c]	FaN[3d]<br>FaN[3e]<br>---[3f]	F-N[3d]<br>F-N[3e]<br>---[3f]	**0**	FaN[3d]<br>FaN[3e]<br>---[3f]
nirBDC-cysG	F-N	F-N	**0**	**0**	**0**	**0**	F-N	F-N	**0**	F-N
dmsABC	f-n	**0**	F-N	F-N	F-N	**0**	**0**	fa-	**0**	fan
torYZ	**0**	--n	**0**	FAN	FAN	FAN	**0**	--N	--N	--N
torCAD	**0**	**0**	fan[4a]	**0**	**0**	**0**	---[4b]<br>Fa-[4b]	---[4b]<br>F-n[4b]	-a-[4b]<br>Fa-[4b]	--n[4b]<br>F--[4b]
frdABCD	-A-	fA-	FAN	FAN	FAN	FAN	faN	--N	--N	faN
fdoGHI	FAN	FAN	FAn	FAn	-A-	**0**	**0**	**0**	**0**	**0**
fdnGHI	**0**	**0**				**0**	**0**	**0**	**0**	**0**
fdhD	-AN	-AN	fAn	fAn	-A-	-A-	**0**	---	**0**	**0**
nqrABCDEF	Fa-	F--	FAN	FAN	FA-	FAN	FA-	FA-	FA-	FAn
ndh	FA-	FAn	**0**	fA-	-An	fAn	F--	F--	F--	F--
glpABC	F--	Fa-	F--	---	F--	Fa-	Fa-	**0**	F--	F-n
glpD	F--	F--	**0**	f--	**0**	**0**	---	---	f--	fa-
dadAX	-A-	-A-	**0**	**0**	**0**	**0**	fA-	-An	-An	**0**
Molibdenum cofactor synthesis										
moaABCDE	Fa-[5a]	F--[5a]	F--[5a]	F-N[5a]	F-N[5a]	FaN[5a]	--N[5b]	--N[5b]	--N[5b]	---[5b]
moeAB	---	f--	F--	FA-	FA-	FA-	---	---	---	-a-
Central metabolism and fermentation										
pgk	---	---	--N	---	faN	-aN	F--	F--	F--	F--
eno	---	-a-	F-N	FaN	F-N	F-	---	--n	---	---
aldB	**0**	**0**	**0**	**0**	**0**	**0**	FA-	FA-	FA-	FA-
adhE	fa-	---	f-n	f-n	**0**	---	FAN	FAN	FAN	FAN
pdhR-aceEF-	FA-[6a]	FA-[6a]	-A-[6b]	-An[6b]	-A-[6b]	-An[6b]	FA-[6a]	F-N[6a]	FAN[6a]	FAN[6a]

lpdA										
pflB	--N	--N	-AN	-AN	-A-	f-N	-AN	--N	fA-	-AN
yfiD	Fa-	F--	FA-	FA-	FA-	-An	F-N	FAN	FAN	FAN
mdh	-A-	fA-	f-N	-aN	f-N	-aN	FA-	FA-	FA-	---
pckA	---	---	--n	-A-	fAn	fA-	-A-	-An	-A-	fAn
ppsA	F--	F--	**0**	**0**	**0**	**0**	-A-	fA-	---	-A-
aspA	f--	-a-	-A-	f--	-A-	-An	--N	--N	0	faN
sfcA	--N	faN	**0**	---	**0**	**0**	F--	F--	F--	F-n
sdhCDAB	-A-	-A-	**0**	**0**	**0**	**0**	-A-	fA-	fA-	-A-
gltA	-A-	-A-	-an	**0**	**0**	**0**	-A-	fA-	fA-	-A-
fumC	f--	---	-aN	--N	--N	f--	**0**	-a-	fa-	**0**
sucABCD	---[7a]	---[7a]	-AN[7a]	-A-[7a]	--N[7b]<br>fAN[7c]	fAN[7a]	---[7a]	---[7a]	---[7a]	-a-[7a]
ldhA	---	f--	**0**	---	-a-	**0**	-a-	--N	f-N	-aN
talB	f--	---	-aN	**0**	f-N	f-N	-a-	f--	---	---
Metabolism of carbohydrates										
mtlADR	fA-	-A-	---	**0**	**0**	**0**	F--	Fa-	F--	**0**
nagBACD	---[8a]	---[8a]	---[8b]<br>fa-[8c]	---[8b]<br>---[8c]	---[8b]	--n[8b]	F--[8d]<br>f--[8e]	F--[8d]<br>f--[8e]	F--[8d]<br>---[8e]	F-n[8d]<br>---[8e]
ptsHI-crr	--n	---	-A-	-An	-A-	fA-	---	--n	-a-	--n
deoCABD	f--[9a]	-a-[9a]	---[9b]	---[9b]	f--[9b]	f--[9c]	--N[9a]	--N[9a]	---[9a]	--N[9a]
malQ-glgBXCAP	---[10a]<br>Fa-[10b]	---[10a]<br>F--[10b]	F-N[10c]	F-N[10c]	F-N[10c]	**0**	--n[10d]<br>-an[10e]<br>---[10f]<br>---[10g]<br>---[10h]	f--[10b]<br>---[10e]<br>---[10f]<br>---[10g]<br>---[10h]	---[10b]<br>---[10e]<br>fa-[10f]<br>---[10g]<br>---[10h]	---[10b]<br>-a-[10e]<br>f--[10f]<br>-an[10g]<br>--n[10h]
Fatty acids metabolism										
fadIJ	-A-	-A-	**0**	**0**	**0**	**0**	FA-	FA-	F--	FA-
fadBA	FAn	FA-	**0**	**0**	**0**	**0**	-A-	fA-	-A-	fA-
fadD	fA-	-An	---	-a-	-an	f--	-A-	-A-	-A-	-A-
acpP-fabF	-A-[11a]	-A-[11a]	fan[11b]	---[11b]	---[11b]	---[11b]	F--[11a]	F--[11a]	F--[11a]	---[11a]
Oxygen stress response										
sodA	-A-	-A-	FA-	FA-	FAn	FA-	**0**	**0**	**0**	**0**
Nucleotide reductases										

nrdDG	F--	F--	f--	-an	f--	**0**	F--	Fa-	F--	F--
nrdAB	--n	---	F-n	F--	Fa-	---	---	---	f--	f--
Transport										
focA	FAN	FAN	FA-	FA-	FA-	FA-	---	f--	---	fa-
dcuB	-A-	fA-	F-N	F-N	**0**	F-N	-a-	---	f--	fan
dcuA	-A-	fA-	--N	f-N	f-N	-a-	---	--n	---	--n
dcuC	**0**	f--	-a-	-a-	**0**	**0**	Fa-	F--	F--	Fa-
gltP	-An	fA-	**0**	**0**	**0**	**0**	fAn	-A-	-A-	fAn
glpFK	-An	-A-	f--	**0**	-an	fa-	-A-	-An	---	-An
gntXY	---	---	FA-	F--	FA-	FA-	---	---	---	--n
fadL	FA-	FA-	---	-a-	f--	-a-	FA-	-A-	FA-	FA-
feoAB	---	f--	**0**	**0**	**0**	**0**	FAN	FAN	-AN	FA-
Peptidase T										
pepT	F--	F--	F--	F--	F--	**0**	F--	Fa-	F--	Fan
Transcription regulators										
fnr	F--	F--	FAN	FAN	FAN	FA-	Fa-	Fa-	F--	F--
arcA	FA-	FA-	f--	---	f-n	-a-	FA-	FA-	FA-	FA-
narQP	-a- [12a]	--n [12a]	f-- [12a]<br>f-- [12b]	--n [12a]<br>--- [12b]	--n [12a]<br>fa- [12b]	--- [12a]<br>-a- [12b]	FAN [12c]	FAN [12c]	FAN [12c]	FAN [12c]
dusB-fis	-a-	---	FA-	-A-	F--	FA-	---	---	---	f--
cpxRA	---	---	FA-	FA-	FA-	FAn	---	---	---	-a-
oxyR	---	---	-A-	-A-	-A-	fA-	---	f--	---	-a-
fur	---	---	F--	Fa-	F--	F--	-A-	-A-	-A-	-An
