# Supplementary material for: Comparative genomic analysis of regulation of anaerobic respiration in ten genomes from three families of gamma-proteobacteria (Enterobacteriaceae, Pasteurellaceae, Vibrionaceae)
Source: BMC Genomics. 2007 Feb 21;8:54. doi: 10.1186/1471-2164-8-54 (PMC1805755; doi:10.1186/1471-2164-8-54)
Supplement: Additional File 2 — Observed changes in the operon structures. For genome abbreviations see "Methods". The superscripts correspond to the superscripts in additional file 1. [file 1471-2164-8-54-S2.pdf]

<table>
<tr><th rowspan="2">Genes</th><th colspan="10">Genome</th></tr>
<tr><th>YP</th><th>YE</th><th>PM</th><th>AA</th><th>HI</th><th>HD</th><th>VV</th><th>VP</th><th>VC</th><th>VF</th></tr>
<tr><td>atpIBEFHAGDC</td><td colspan="2">[1a] atpIBEFHAGDC</td><td colspan="4">[1b] atpBEFHAGDC</td><td colspan="4">[1a] atpIBEFHAGDC</td></tr>
<tr><td>napFDAGHBC</td><td colspan="2">[2a] napFDABC</td><td colspan="4">[2b] napFDAGHBC</td><td colspan="2">[2a] napFDABC
[2c] napGH</td><td colspan="2">[2a] napFDABC</td></tr>
<tr><td>nrfABCDEXFG</td><td>0</td><td>0</td><td colspan="2">[3a] nrfABCDEXFG</td><td colspan="2">[3b] nrfABCD
[3c] nrfEXFG</td><td colspan="2">[3d] nrfA
[3e] nrfBCDEXF
[3f] nrfG</td><td>0</td><td>[3d] nrfA
[3d] nrfBCDEXF
[3f] nrfG</td></tr>
<tr><td>torCAD</td><td>0</td><td>0</td><td>[4a] torCAD</td><td>0</td><td>0</td><td>0</td><td colspan="4">[4b] torCA
[4c] torD</td></tr>
<tr><td>moaABCDE</td><td colspan="2">[5a] moaACDE</td><td colspan="4">[5a] moaACDE</td><td colspan="4">[5b] moaABCDE</td></tr>
<tr><td>pdhR-aceEF-lpdA</td><td colspan="2">[6a] pdhR-aceEF-lpdA</td><td colspan="4">[6b] aceEF-lpdA</td><td colspan="4">[6a] pdhR-aceEF-lpdA</td></tr>
<tr><td>sucABCD</td><td colspan="2">[7a] sucABCD</td><td colspan="2">[7a] sucABCD</td><td>[7b] sucAB
[7c] sucCD</td><td>[7a] sucABCD</td><td colspan="4">[7a] sucABCD</td></tr>
<tr><td>nagBACD</td><td colspan="2">[8a] nagBACD</td><td colspan="2">[8b] nagBA
[8c] nagC</td><td colspan="2">[8b] nagBA</td><td colspan="4">[8d] nagB
[8e] nagAC</td></tr>
<tr><td>deoCABD</td><td colspan="2">[9a] deoCABD</td><td colspan="3">[9b] deoD</td><td>[9c] deoCD</td><td colspan="4">[9a] deoCABD</td></tr>
<tr><td>malQ-glgBXCAP</td><td colspan="2">[10a] malQ
[10b] glgBXCAP</td><td colspan="3">[10c] malQ-glgBXCAP</td><td>0</td><td colspan="4">[10d] malQ
[10e] glgB
[10f] glgX
[10g] glgC
[10h] glgA</td></tr>
<tr><td>acpP-fabF</td><td colspan="2">[11a] acpP-fabF</td><td colspan="4">[11b] acpP</td><td colspan="4">[11a] acpP-fabF</td></tr>
<tr><td>narQP</td><td colspan="2">[12a] narP</td><td colspan="4">[12a] narP
[12b] narQ</td><td colspan="4">[12c] narQP</td></tr>
</table>

Description of additional data files

Ravcheev_Gerasimova_Add1.pdf
PDF file.
Title of data: Phylogenetic trees for FdnG/FdoG (a), FdnH/FdoH (b) and FdnI/FdoI.
Description of data: The trees were constructed by the neighbour-joining method. The expected fraction of amino acid substitutions is indicated for branches longer than 0.05. Genome abbreviations: EC – *Escherichia coli*, ST – *Salmonella typhi*, EO – *Erwinia carotovora,* YP – *Yersinia pestis*, YE – *Y. enterocolitica*, PM – *Pasteurella multocida*, AA – *Actinobacillus actinomycetemcomitans*, HI – *Haemophilus influenzae*.
The analysis of the trees indicates that the most parsimonious evolutionary scenario is the duplication of the fumarate dehydrogenase operon in the ancestral Enterobacteria and further species-specific loss of the Fdn copy in the *Yersinia* lineage. In any case, it is clear that the fumarate dehydrogenase of the *Yersinia* spp. is Fdo.

Ravcheev_Gerasimova_Add2.pdf
PDF file.
Title of data: Additional predicted regulatory interactions
Description of data: "For genome abbreviations see "Materials and Methods". Candidate sites are shown by letters: F – Fnr sites, A – ArcA sites, N – NarP sites; conserved sites are shown by capital letters, non-conserved ones, by lower-case letters (for details see "Materials and Methods"). Absence of the corresponding sites is shown by dashes. Absent genes are shown by zeros. Superscripts point to changes in operon structures described in detail in additional file 3. $^{\#}$: operon forms a divergon with an another one."

Ravcheev_Gerasimova_Add3.pdf
PDF file.
Title of data: Additional observed changes in the operon structures
Description of data: For genome abbreviations see "Materials and Methods". Superscripts coincide with the corresponding superscripts in additional file 2.

Ravcheev_Gerasimova_Add4.pdf
PDF file.
Title of data: False-positive predictions
Description of data: The last column lists the reasons to exclude the operons. For genome abbreviations see "Materials and Methods".

Ravcheev_Gerasimova_Add5.pdf
PDF file.
Title of data: Phylogenetic trees for NarL/NarP (a) and NarX/NarQ (b).
Description of data: The trees were constructed by the neighbour-joining method. The expected fraction of amino acid substitutions is indicated for brances. The branches corresponding to *Yersinia* spp. proteins are shown with broken lines. Genome abbreviations: EC – *Escherichia coli*, ST – *Salmonella typhi*, EO – *Erwinia carotovora,* YP – *Yersinia pestis*, YE – *Y. enterocolitica*, PM – *Pasteurella multocida*, AA – *Actinobacillus actinomycetemcomitans*, HI – *Haemophilus influenzae*, HD – *Haemophilus ducreyi*, VV – *Vibrio vulnificus*, VP – *V. parahaemolyticus*, VC – *V. cholerae*, VF – *Vibrio fischeri*.

Ravcheev_Gerasimova_Add6.pdf
PDF file.
Title of data: Position weight matrices (profiles) for Fnr (a), ArcA (b) and NarP (c) binding sites
Description of data: Rows: nucleotides. Columns: Positions. Cells: positional nucleotide weights.

Ravcheev_Gerasimova_Add7.pdf
PDF file.
Title of data: Sequence logos for the Fnr (a), ArcA (b) and NarP (c) binding sites

Description of data: Horizontal axis, position in the binding site; vertical axis, information content in bits. The height of each column is proportional to the positional information content in the given position; the height of each individual symbol reflects its prevalence in the given position.
