# Supplementary figures and images for: Comparative genomic analysis of regulation of anaerobic respiration in ten genomes from three families of gamma-proteobacteria (Enterobacteriaceae, Pasteurellaceae, Vibrionaceae)
Source: BMC Genomics. 2007 Feb 21;8:54. doi: 10.1186/1471-2164-8-54 (PMC1805755; doi:10.1186/1471-2164-8-54)

**(a)**

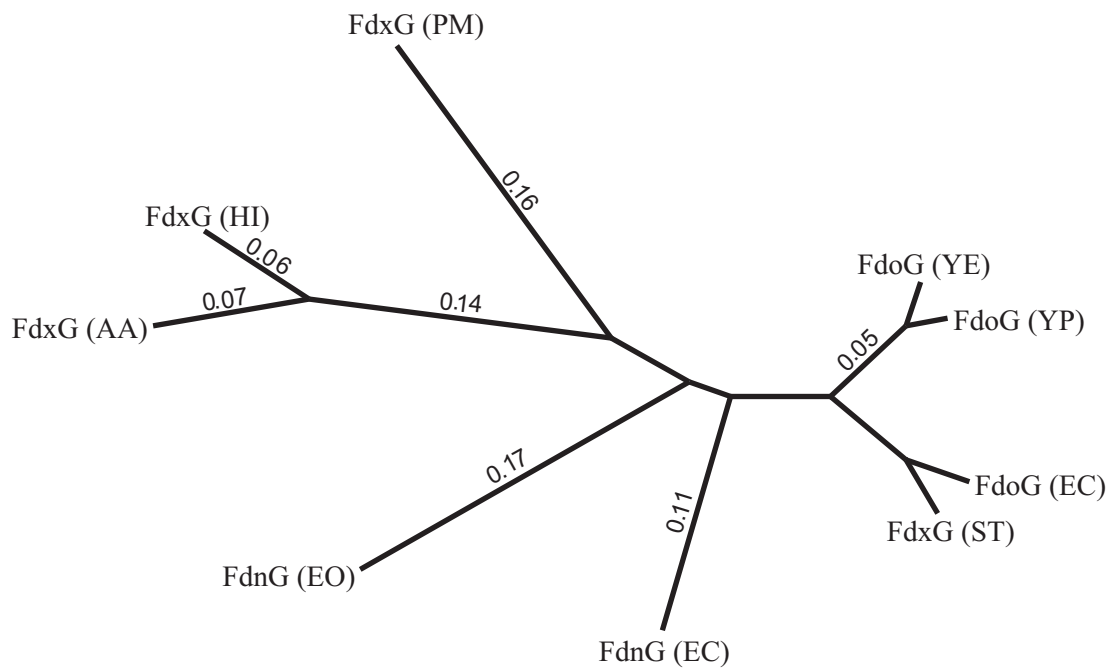

**(b)**

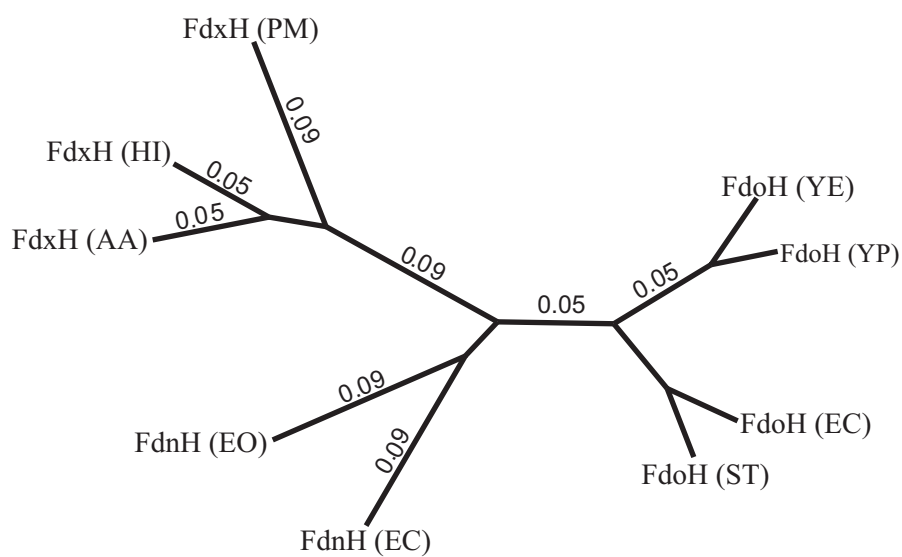

**(c)**

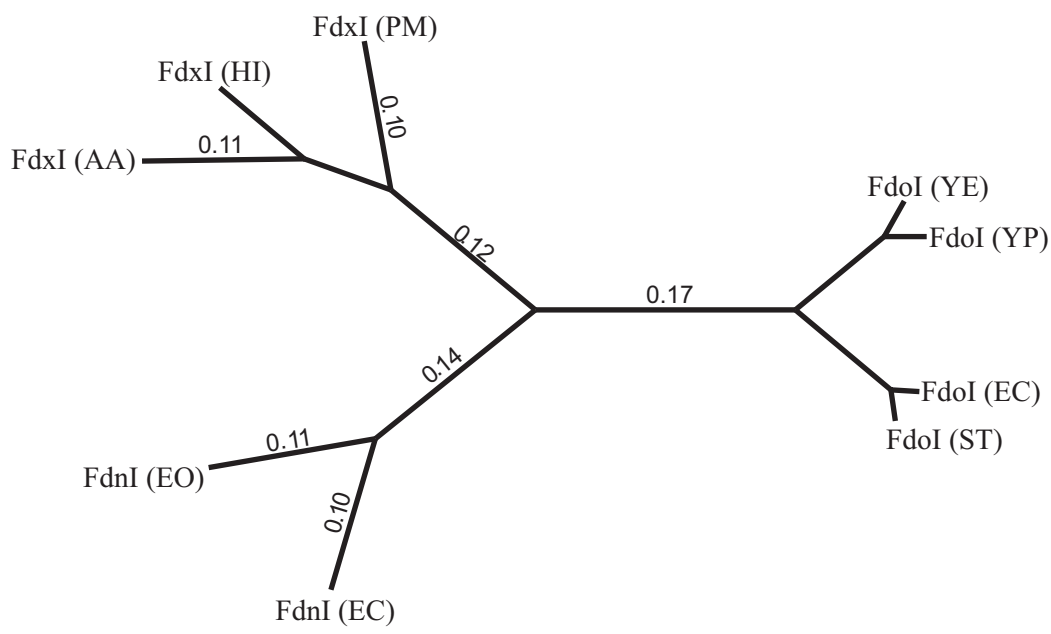

Supplement: Additional File 3 — Phylogenetic trees for FdnG/FdoG (a), FdnH/FdoH (b) and FdnI/FdoI. The trees were constructed by the neighbour-joining method. The expected fraction of amino acid substitutions is indicated for branches longer than 0.05. Genome abbreviations: EC – Escherichia coli, ST – Salmonella typhi, EO – Erwinia carotovora, YP – Yersinia pestis, YE – Y. enterocolitica, PM – Pasteurella multocida, AA – Actinobacillus actinomycetemcomitans, HI – Haemophilus influenzae. The analysis of the trees indicates that the most parsimonious evolutionary scenario is the duplication of the fumarate dehydrogenase operon in the ancestral Enterobacteria and further species-specific loss of the Fdn copy in the Yersinia lineage. In any case, it is clear that the fumarate dehydrogenase of the Yersinia spp. is Fdo. [file 1471-2164-8-54-S3.pdf]

**(a)**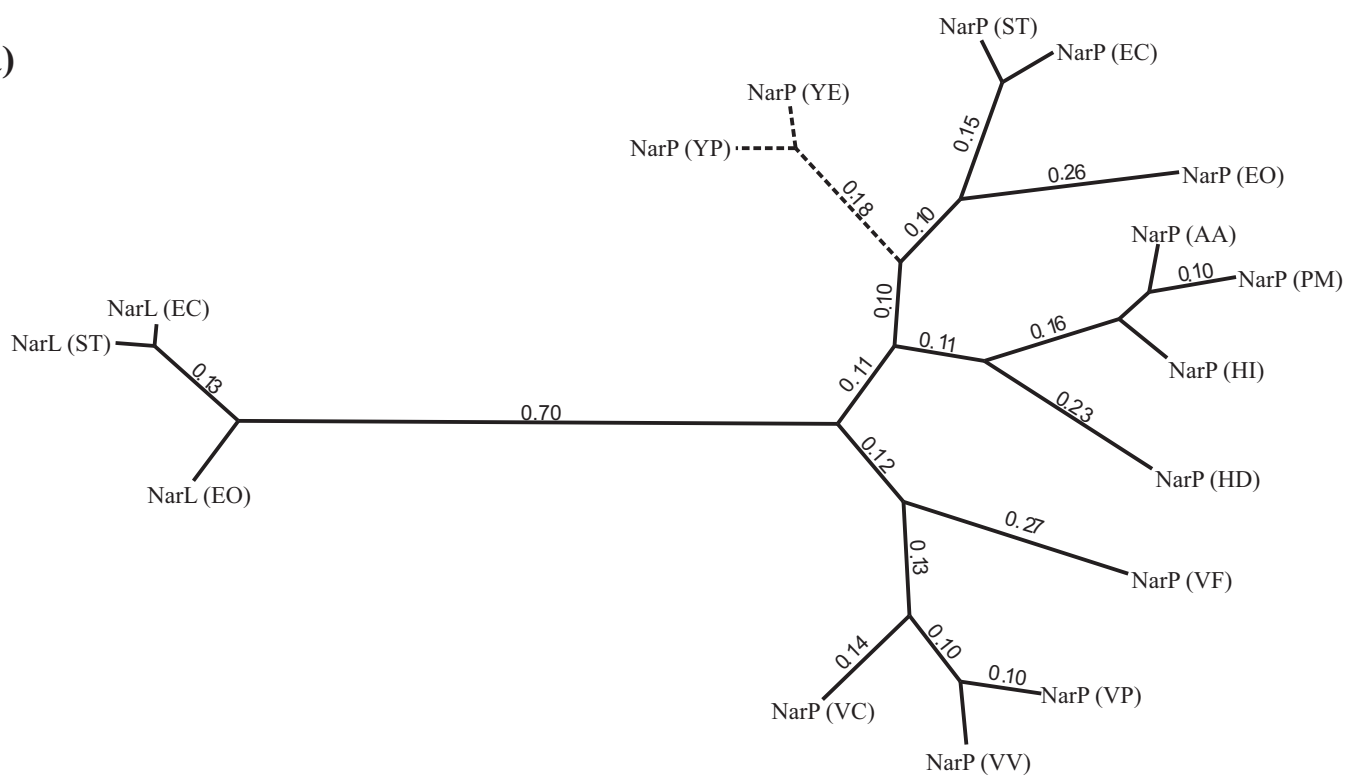**(b)**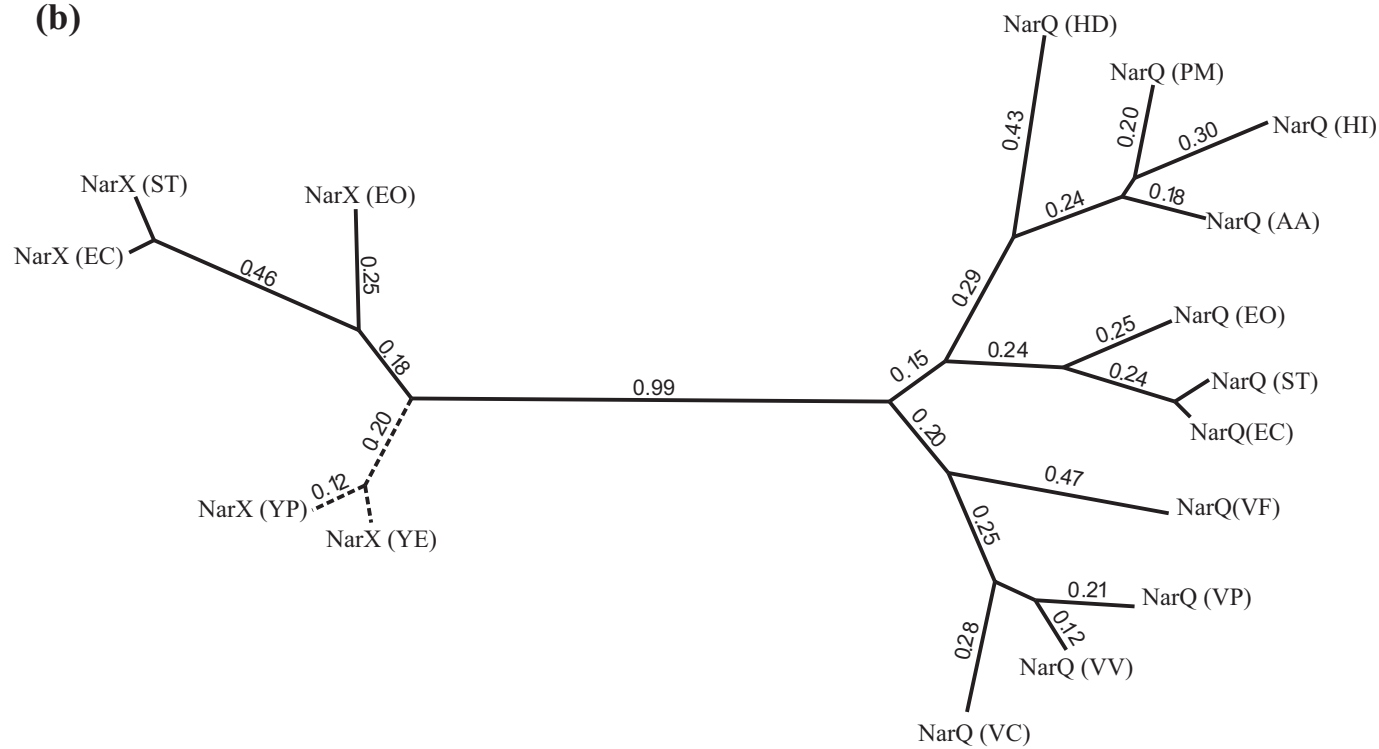

Supplement: Additional File 4 — Additional predicted regulatory interactions. "For genome abbreviations see "Methods". Candidate sites are shown by letters: F – Fnr sites, A – ArcA sites, N – NarP sites; conserved sites are shown by capital letters, non-conserved ones, by lower-case letters (for details see "Methods"). Absence of the corresponding sites is shown by dashes. Absent genes are shown by zeros. Superscripts point to changes in operon structures described in detail in additional file 5. #: operon forms a divergon with an another one." [file 1471-2164-8-54-S4.pdf]
