# Supplementary material for: Comparative genomic analysis of regulation of anaerobic respiration in ten genomes from three families of gamma-proteobacteria (Enterobacteriaceae, Pasteurellaceae, Vibrionaceae)
Source: BMC Genomics. 2007 Feb 21;8:54. doi: 10.1186/1471-2164-8-54 (PMC1805755; doi:10.1186/1471-2164-8-54)
Supplement: Additional File 5 — Additional observed changes in the operon structures. For genome abbreviations see "Methods". Superscripts coincide with the corresponding superscripts in additional file 4. [file 1471-2164-8-54-S5.pdf]

Operon	Genome									
	**YP**	**YE**	**PM**	**AA**	**HI**	**HD**	**VV**	**VP**	**VC**	**VF**
	**Regulatory sites**									
Lytic murein transglycosylase										
mltA	--N	--N	---	---	---	-a-	---	fA-	fA-	-A-
Metabolism of nucleotides										
pyrG	---	---	F--	---	F--	Fa-	F--	F--	F--	Fa-
ndk	-A-	-A-	-A-	-A-	-A-	---	---	---	---	---
Transport										
nhaB-dsbB[#]	-A-	fA-	-A-	-A-	f--	fA-	-A-	---	fAn	fA-
yhdWXYZ[#]	**0**	--n	**0**	**0**	**0**	**0**	-A-	-A-	-A-	fA-
brnQ	---	---	---	---	-a-	-a-	-A-	fA-	fAn	-A-
mtr	---	f--	--N	---	faN	-aN	**0**	**0**	**0**	**0**
yhjX	**0**	---	**0**	**0**	**0**	-a-	-A-	-A-	-A-	---
Heat shock shaperones										
dnaKJ	FA-	FA-	fA-	-A-	--n	-A-	-A-	fA-	-A-	-A-
Peptidyl-prolyl cis-trans isomerase										
fkpA[#]	---	---	f-N	--N	--N	---	---	f--	-an	f--
Transcription regulators										
fadR[#]	-A-	fA-	-A-	-A-	f--	fA-	FA-	---	FAn	FA-
gcvA	--n	---	--N	--N	-aN	fa-	---	---	---	f--
torR[#]	**0**	**0**	**0**	**0**	**0**	**0**	Fa-	F-n	Fa-	F--
sgrR	---	---	**0**	**0**	**0**	**0**	--N	f-N	f-N	---
Metabolism of folates and one-carbon compounds										
yjjPB-folA	--n [13a]	--- [13a]	f-N [13a]	--N [13a]	f-N [13a]	--N [13b]	--- [13a]	-a- [13a]	-an [13a]	f-- [13a]
glyA	---	--n	-a-	--n	---	0	FA-	FA-	FA-	F--
Heme metabolism										
hemE	fa-	---	---	fa-	**0**	**0**	-A-	-A-	-A-	-A-
Genes for sulfur metabolism										

cysJIH	---	---	**0**	**0**	**0**	**0**	--N	--N	---	--N
Metabolism of aminoacids										
argD	---	---	---	**0**	**0**	---	-A-	-A-	fA-	fA-
aroH	--n	-a-	**0**	**0**	**0**	**0**	-A-	-A-	-A-	-A-
Biotin metabolism										
bioABFCD	F-- [14a]<br>F-- [14b]	F-- [14a]<br>F-- [14b]	-a- [14c]<br>--- [14d]<br>F-- [14e]	--N [14c]<br>f-- [14d]<br>F-- [14e]	--N [14c]<br>--- [14d]<br>Fa- [14e]	f-N [14c]<br>-a- [14d]<br>Fan [14e]	f-- [14a]<br>f-- [14b]	f-- [14a]<br>f-- [14b]	--- [14a]<br>--- [14b]	--- [14a]<br>--- [14b]
DNA repair										
VV12876-75-74-tag	--- [15a]	--- [15a]	-A- [15a]	fA- [15a]	-A- [15a]	**0**	-A- [15b]	-An [15b]	-A- [15b]	-A- [15b]
Colicin transpot system										
ompW	F-N	F-N	fa-	-an	**0**	**0**	**0**	f--	f--	**0**
tolB-pal-ybgF	-A-	fA-	f--	-A-	-An	-A-	-a-	---	-a-	---
RNA helicase										
srmB	---	---	---	---	---	fa-	-A-	fA-	fA-	-A-
Unknown function										
VV12099	**0**	**0**	**0**	**0**	**0**	**0**	F-N	F-N	Fa-	FaN
mrp	F--	F--	---	--N	f-N	f-N	---	---	f--	f--
yfiO	F--	F--	---	---	---	-a-	-A-	---	fA-	-A-
yhbUV	fa-	---	**0**	**0**	**0**	**0**	--N	--N	--N	--N
VV12702 #	**0**	**0**	**0**	**0**	**0**	**0**	-A-	-A-	-A-	fA-
yjcE	-a-	f--	---	**0**	**0**	**0**	-A-	-A-	-A-	fA-
yfiC #	---	---	---	---	---	-a-	-A-	fA-	fAn	-A-
VV10415	**0**	**0**	**0**	**0**	**0**	**0**	-A-	-A-	fA-	-A-
VV11834	**0**	**0**	**0**	**0**	**0**	**0**	fA-	-A-	fA-	-A-
slyX #	---	---	--N	--N	--N	---	---	f--	-a-	**0**
yhjA	f--	**0**	-an	f-n	**0**	**0**	F--	F--	F--	-a-
slyB	f--	-a-	-A-	---	-A-	-A-	---	---	**0**	**0**
VV12768-67-66-65	**0**	**0**	**0**	**0**	**0**	**0**	-A-	-A-	-A-	**0**
VV12695	**0**	**0**	---	---	f--	---	-A-	-A-	-An	---
VV20742	**0**	**0**	**0**	**0**	**0**	**0**	-A-	-An	-A-	**0**

VV12832	**0**	**0**	**0**	**0**	**0**	**0**	-An	-A-	-A-	**0**
VV12996	**0**	**0**	-a-	---	---	**0**	-A-	---	-A-	fA-
VV11084	**0**	**0**	**0**	**0**	**0**	**0**	-A-	-A-	-A-	**0**
