# Supplementary material for: Comparative genomic analysis of regulation of anaerobic respiration in ten genomes from three families of gamma-proteobacteria (Enterobacteriaceae, Pasteurellaceae, Vibrionaceae)
Source: BMC Genomics. 2007 Feb 21;8:54. doi: 10.1186/1471-2164-8-54 (PMC1805755; doi:10.1186/1471-2164-8-54)
Supplement: Additional File 6 — False-positive predictions. The last column lists the reasons to exclude the operons. For genome abbreviations see "Methods". [file 1471-2164-8-54-S6.pdf]

Genes	Genome									
	**YP**	**YE**	**PM**	**AA**	**HI**	**HD**	**VV**	**VP**	**VC**	**VF**
yjjPB-folA	[13a] *yjjPB-folA*		[13a] *yjjPB-folA*			[13b] *folA*	[13a] *yjjPB-folA*			
bioABFCD	[14a] *bioA*<br>[14b] *bioBFCD*		[14c] *bioAFHCD*$_2$<br>[14d] *bioB*<br>[14e] *bioD*				[14a] *bioA*<br>[14b] *bioBFCD*			
VV12876-75-74-tag	[15a] *tag*		[15a] *tag*			**0**	[15b] *VV12876-75-74-tag*			
