# Supplementary material for: Comparative genomic analysis of regulation of anaerobic respiration in ten genomes from three families of gamma-proteobacteria (Enterobacteriaceae, Pasteurellaceae, Vibrionaceae)
Source: BMC Genomics. 2007 Feb 21;8:54. doi: 10.1186/1471-2164-8-54 (PMC1805755; doi:10.1186/1471-2164-8-54)
Supplement: Additional File 7 — Phylogenetic trees for NarL/NarP (a) and NarX/NarQ (b). The trees were constructed by the neighbour-joining method. The expected fraction of amino acid substitutions is indicated for brances. The branches corresponding to Yersinia spp. proteins are shown with broken lines. Genome abbreviations: EC – Escherichia coli, ST – Salmonella typhi, EO – Erwinia carotovora, YP – Yersinia pestis, YE – Y. enterocolitica, PM – Pasteurella multocida, AA – Actinobacillus actinomycetemcomitans, HI – Haemophilus influenzae, HD – Haemophilus ducreyi, VV – Vibrio vulnificus, VP – V. parahaemolyticus, VC – V. cholerae, VF – Vibrio fischeri. [file 1471-2164-8-54-S7.pdf]

Operon	Genome										Reasons
	YP	YE	PM	AA	HI	HD	VV	VP	VC	VF	
	Regulatory sites										
argR	-A-	fA-	--N	-aN	f-N	-aN	FA-	FA-	FA-	---	Forms a divergon with the *mdh* gene in all studied genomes.
ung	Fa-	F--	FA-	FA-	FA-	-A-	---	---	---	f--	Forms a divergon with *yfiD* in *Yersinia spp* and Pasteurellaceae. In other genomes sites upstream of *ung* were not found.
nfo	---	---	0	0	0	-a-	F-n	FA-	FAn	FAn	Forms a divergon with *yfiD* in Vibrionaceae. In other genomes sites upstream of *nfo* were not found.
potABCD	0	0	F--	F--	F--	Fan	---	---	---	---	Forms a divergon with *pepT* in Pasteurellaceae. In other genomes sites upstream of the *pot* operon were not found.

Supplementary figures

Figure 1S. Position weight matrices (profiles) for Fnr (A), ArcA (B) and NarP (C) binding sites. Each column shows the weights of the given nucleotide in the consecutive positions of the respective binding signal.

A

Fnr

A	C	T	G
-0.09	-0.09	-0.32	0.49
-0.29	0.05	-0.29	0.52
-0.26	-0.26	0.55	-0.03
0.40	-0.15	-0.37	0.12
-0.39	0.07	-0.06	0.38
-0.08	-0.01	0.02	0.07
0.07	-0.26	0.00	0.19
0.19	0.00	-0.26	0.07
0.07	0.02	-0.01	-0.08
0.38	-0.06	0.07	-0.39
0.12	-0.37	-0.15	0.40
-0.03	0.55	-0.26	-0.26
0.52	-0.29	0.05	-0.29
0.49	-0.32	-0.09	-0.09

B

ArcA

A	C	T	G
-0.17	-0.17	-0.17	0.50
0.41	-0.22	-0.22	0.03
0.41	-0.22	-0.22	0.03
-0.17	0.50	-0.17	-0.17
0.50	-0.17	-0.17	-0.17
0.15	-0.31	-0.05	0.21
0.13	-0.25	-0.25	0.36
-0.04	-0.30	0.08	0.26
0.26	-0.04	-0.30	0.08
0.15	-0.31	0.21	-0.05
-0.17	-0.17	-0.17	0.50
0.03	-0.22	-0.22	0.41
0.41	0.03	-0.22	-0.22
0.50	-0.17	-0.17	-0.17
-0.17	0.50	-0.17	-0.17

C

NarP

A	C	T	G
-0.25	0.06	-0.25	0.44
0.38	-0.08	-0.31	0.00
0.17	0.33	-0.17	-0.33
-0.16	0.36	-0.32	0.13
0.07	0.10	-0.40	0.23
0.07	0.30	-0.37	0.00
-0.07	0.02	-0.23	0.29
0.22	-0.18	-0.34	0.30
0.30	-0.34	-0.18	0.22
0.29	-0.23	0.02	-0.07
0.00	-0.37	0.30	0.07
0.23	-0.40	0.10	0.07
0.13	-0.32	0.36	-0.16
-0.33	-0.17	0.33	0.17
0.00	-0.31	-0.08	0.38
0.44	-0.25	0.06	-0.25

Figure 2S. Sequence logos for the Fnr (A), ArcA (B) and NarP (C) binding sites. Horizontal axis, position in the binding site; vertical axis, information content in bits. The height of each column is proportional to the positional information content in the given position; the height of each individual symbol reflects its prevalence in the given position.

A

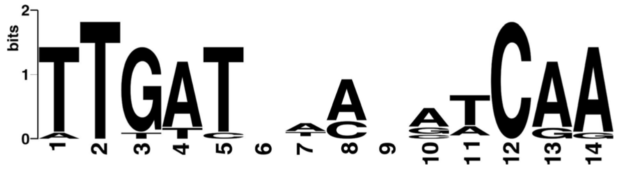

B

C
