# Supplementary material for: Comparative genomic analysis of regulation of anaerobic respiration in ten genomes from three families of gamma-proteobacteria (Enterobacteriaceae, Pasteurellaceae, Vibrionaceae)
Source: BMC Genomics. 2007 Feb 21;8:54. doi: 10.1186/1471-2164-8-54 (PMC1805755; doi:10.1186/1471-2164-8-54)
Supplement: Additional File 8 — Position weight matrices (profiles) for Fnr (a), ArcA (b) and NarP (c) binding sites. Rows: nucleotides. Columns: Positions. Cells: positional nucleotide weights. [file 1471-2164-8-54-S8.pdf]

Position weight matrices (profiles) for Fnr (a), ArcA (b) and NarP (c) binding sites

Each column shows the weights of the given nucleotide in the consecutive positions of the respective binding signal.

(a)

Fnr			
A	C	G	T
-0.09	-0.09	-0.32	0.49
-0.29	0.05	-0.29	0.52
-0.26	-0.26	0.55	-0.03
0.40	-0.15	-0.37	0.12
-0.39	0.07	-0.06	0.38
-0.08	-0.01	0.02	0.07
0.07	-0.26	0.00	0.19
0.19	0.00	-0.26	0.07
0.07	0.02	-0.01	-0.08
0.38	-0.06	0.07	-0.39
0.12	-0.37	-0.15	0.40
-0.03	0.55	-0.26	-0.26
0.52	-0.29	0.05	-0.29
0.49	-0.32	-0.09	-0.09

(b)

ArcA			
A	C	G	T
-0.17	-0.17	-0.17	0.50
0.41	-0.22	-0.22	0.03
0.41	-0.22	-0.22	0.03
-0.17	0.50	-0.17	-0.17
0.50	-0.17	-0.17	-0.17
0.15	-0.31	-0.05	0.21
0.13	-0.25	-0.25	0.36
-0.04	-0.30	0.08	0.26
0.26	-0.04	-0.30	0.08
0.15	-0.31	0.21	-0.05
-0.17	-0.17	-0.17	0.50
0.03	-0.22	-0.22	0.41
0.41	0.03	-0.22	-0.22
0.50	-0.17	-0.17	-0.17
-0.17	0.50	-0.17	-0.17

(c)

NarP			
A	C	G	T
-0.25	0.06	-0.25	0.44
0.38	-0.08	-0.31	0.00
0.17	0.33	-0.17	-0.33
-0.16	0.36	-0.32	0.13
0.07	0.10	-0.40	0.23
0.07	0.30	-0.37	0.00
-0.07	0.02	-0.23	0.29
0.22	-0.18	-0.34	0.30
0.30	-0.34	-0.18	0.22
0.29	-0.23	0.02	-0.07
0.00	-0.37	0.30	0.07
0.23	-0.40	0.10	0.07
0.13	-0.32	0.36	-0.16
-0.33	-0.17	0.33	0.17
0.00	-0.31	-0.08	0.38
0.44	-0.25	0.06	-0.25
