# Supplementary material for: Comparative genomic analysis of regulation of anaerobic respiration in ten genomes from three families of gamma-proteobacteria (Enterobacteriaceae, Pasteurellaceae, Vibrionaceae)
Source: BMC Genomics. 2007 Feb 21;8:54. doi: 10.1186/1471-2164-8-54 (PMC1805755; doi:10.1186/1471-2164-8-54)

Sequence logos for the Fnr (a), ArcA (b) and NarP (c) binding sites

Horizontal axis, position in the binding site; vertical axis, information content in bits. The height of each column is proportional to the positional information content in the given position; the height of each individual symbol reflects its prevalence in the given position.

(a)

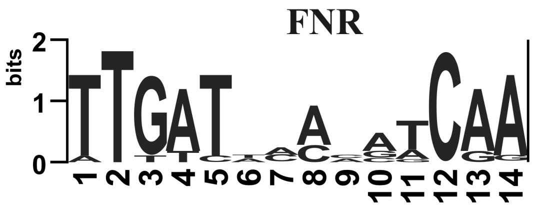

(b)

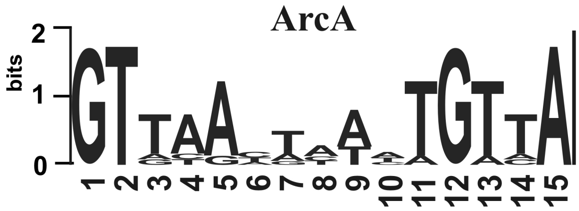

(c)

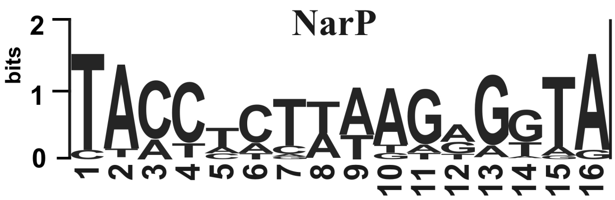

Supplement: Additional File 9 — Sequence logos for the Fnr (a), ArcA (b) and NarP (c) binding sites. Horizontal axis, position in the binding site; vertical axis, information content in bits. The height of each column is proportional to the positional information content in the given position; the height of each individual symbol reflects its prevalence in the given position. [file 1471-2164-8-54-S9.pdf]
